# A Bullet with Low Velocity

**Published:** 1899-12-09

**Authors:** 


### A BULLET WITH LOW VELOCITY.

In curious contrast with these results is a case recorded by Mr. Barker in the same journal, in which a man fired two shots from a revolver into his mouth. One of the bullets seems to have lodged extracranially, but the other, after passing through the palate and the sphenoid, had apparently impinged on the inner surface of the calvarium and fallen back into the brain, where it lodged for a considerable time without doing much harm. After about four weeks, however, a condition of partial hemiplegia supervened, and ultimately he suffered from a succession of convulsions attended by rise of temperature. On these symptoms manifesting themselves Mr. Barker, on the sixty-ninth day after the injury, performed an ingeniously devised operation and removed the bullet from the brain with very good effect. The point of interest, however, to which we would draw attention is the contrast between the ghastly effects of bullets at a high velocity and the very trivial immediate symptoms which followed in this case, in which the velocity of the bullet was probably small to begin with and was much diminished by its passage through the palate and sphenoid before it entered the brain. It is quite clear that so far as the rest of the brain was concerned none of the disruptive or explosive effects occurred at all. After the injury he had walked to the pond on Hampstead Heath, where he had washed his face and hands, and had then walked to the police-station to give himself up. Very curiously the patient, in describing some time afterwards what had occurred, stated that on firing the first shot he felt a distinct blow upon the vertex. Putting his hand upon the middle of the vertex he said, "It regularly rattled there."

